# A Reliable and Simple Voltammetric Method for Analysis of Brilliant Blue FCF

**DOI:** 10.3390/s25206424

**Published:** 2025-10-17

**Authors:** Marek Szlósarczyk, Robert Piech, Bartłomiej Pach, Mariusz Stolarczyk, Urszula Hubicka

**Affiliations:** 1 Department of Inorganic Chemistry and Pharmaceutical Analytics, Medical College, Jagiellonian University, Medyczna 9, 30-688 Kraków, Polandmariusz.stolarczyk@uj.edu.pl (M.S.); urszula.hubicka@uj.edu.pl (U.H.); 2 Faculty of Materials Science and Ceramics, AGH University of Krakow, Al. Mickiewicza 30, 30-059 Kraków, Poland; rpiech@agh.edu.pl

**Keywords:** brilliant blue FCF, foodstuff analysis, food additives, mercury film electrodes, voltammetry, UV-Vis spectrophotometry

## Abstract

Synthetic food colourings are widely used because they are stable, inexpensive, reliable, and effective in shaping consumer perception and behaviour, even though some are under scrutiny for adverse health effects. In this work, we present a new sensitive voltammetric method for the determination of brilliant blue FCF (BB) using a cyclic renewable silver-based mercury film electrode (Hg(Ag)FE). The experimental parameters, including pulse height, step potential, preconcentration potential and duration, and the composition of the supporting electrolyte, were systematically optimised. Under these conditions, the calibration curve exhibited linearity within the range of 0.7 up to 250 µg L^−1^. For an Hg(Ag)FE with a surface area of 10.9 mm^2^, with a short preconcentration step of 15 s, the limits of detection (LOD) and quantification (LOQ) of BB were 0.24 µg L^−1^ and 0.72 µg L^−1^, respectively. The repeatability of the method at a concentration level of the analyte as low as 2.0 µg L^−1^, expressed as RSD, was 2.39% (*n* = 6). The proposed method was successfully applied in the analysis of brilliant blue FCF in popular beverages and artificial juices. The obtained results not only verify that BB levels are within acceptable limits, but also enrich the limited data on the quantitative compositions of ‘popular’ beverages.

## 1. Introduction

Food additives have a long history of use, and among them, food colourants are a good example. In the broadest sense, according to the FDA, a food colourant is ‘any dye, pigment or substance which when added or applied to a food, drug or cosmetic, or to the human body, is capable (alone or through reactions with other substances) of imparting color’ (FDA, 2016). One of the most relevant organoleptic attributes is colour, which directly affects consumers’ acceptance and selection of foods, particularly in the case of children [1,2,3]. In addition, there are many reasons for using synthetic pigments for food colouring, such as restoring colour lost during food processing [1,4], standardisation of product appearance [1,5], association of colour with flavour and identity [6], cost-effectiveness, and better stability [1,7,8]. Synthetic colourants are widely used, despite their potential hazards to human health, with brilliant blue FCF (For Colouring Food) being a notable example.

Brilliant Blue FCF (BB), also known as E133, Acid Blue 9, CI 42090, and FD&C Blue No. 1, is a triarylmethane dye that is authorised as a food additive in the EU. It was previously evaluated by two committees: the Joint FAO/WHO Expert Committee on Food Additives (JECFA) in 1970, and the EU Scientific Committee for Food (SCF) in 1975. BB is poorly absorbed and remains mainly unchanged in the faeces, according to available data on its absorption, distribution, metabolism, and excretion [9], but has the ability to cross the blood–brain barrier. Nevertheless, side effects such as allergies, asthma, DNA damage, carcinogenesis, and genotoxicity in humans, as well as a relationship with behavioural disorders, have been reported [1,2,10,11,12,13]. Moreover, blue is a rare colour in food, so we assume it must be artificial. However, this does not reduce the popularity of products containing synthetic blue dye [3,14,15]. Note also that blue colour in food is popular among young people due to a mix of novelty, flavour expectations, marketing, and cultural associations [3,16,17]. E133 is one of the most widely used synthetic blue colourings in beverages, including isotonic (sports) drinks, energy drinks, and some alcoholic beverages, often in combination with tartrazine.

The labelling of food products is required by international standards for all food colour additives, but does not impose an obligation to state the exact amount of the additive [18,19,20]. Moreover, manufacturers must include an additional warning, ‘may have an adverse effect on activity and attention in children’, when BB is present in a product. The regulation of the average daily intake of brilliant blue FCF is set at 6 mg/kg b.w. [9]. This requires the quantitative determination of synthetic food colourings such as BB to ensure food safety.

The analytical methods most often used to determine BB in different foodstuffs are spectrophotometry [21] preceded by an extraction step, which vary in terms of sophistication [22,23]; capillary electrophoresis [24]; thin-layer chromatography (TLC) [19,25]; and high-performance thin-layer chromatography (HPTLC) [26], and assessing a high variety of food matrices is not generally feasible. Generally, the colorants are extracted, purified, and concentrated prior to analysis, even when using liquid chromatography (LC) systems [27]. Various purification techniques, such as liquid–liquid extraction [28] and solid-phase extraction [29], have been employed for synthetic colorants. However, these methods often involve substantial use of toxic solvents and synthetic reagents, leading to increased costs and negative environmental impacts. In these methods, sample preparation is complex, time-consuming, and error-prone, and the solvent system contains toxic chemicals.

A different approach to analysis is electrochemical techniques, which have been commonly based on the reduction and oxidation signals of BB. Among these techniques, electrochemical detection is a more attractive option because it is inexpensive, highly sensitive, and easy to miniaturize. Electrochemical methods, such as voltammetry, have been widely applied for the determination of dyes [30,31,32,33]. In this field, there are two main methods of electrode fabrication based on the glassy carbon electrode (GCE) and carbon paste electrode (CPE) types. In the case of GCEs, the electrode surface is modified by a widely used “drop-casting” technique to increase the surface area and amplify the signal. The modifying layer is composed of nanoparticles, such as multiwalled carbon nanotubes [34], MoS_2_-PVP particles [35], and MnO_2_ nanorods [33], for chemical sensing as well as material evaluation. In the case of the CPE type, the material for electrode fabrication, a mixture of dispersed solid modifier particles such as CuNiFe_2_O_4_ nanospheres [36] or CuO nanoparticles [37] with mineral (parafin) oil, is added to a polyethylene tube in the form of a paste or can be pressurized with polyethylene. This type of electrode can be modified in a similar way to the GCE surface. However, the finishing of the surface should be performed by perfectly uniformly polishing, forming, or cutting the edge. Afterwards, modification of the electrode surface is the second most critical part of the fabrication process, involving synthesis of modified nanoparticles [35,36,37], preparation of a proper dispersion to resolve aggregation problems [33] and heterogeneity, or carrying out a layer-by-layer complex electropolymerization process [37]. Finally, the electrode should be activated and manually or electrochemically cleaned after a series of measurements. However, a problem known as ‘coffee ring’ and its related effects may occur on the surface, causing significant limitations to the reproducibility of drop-casted surfaces [38]. Additionally, the electrode film on the chemically modified electrodes has a fixed surface area, and is obviously significantly different from its original form. Therefore, concerning the above information, it is of great importance to search for novel electrodes with high sensitivity for the determination of BB FCF, but sensor fabrication must be simplified for application to these working electrodes.

The proposed electrodes are not easy to use, require careful preparation, and have a heterogeneous and limited surface area, and their operating range is mainly limited to positive potential. In contrast, the renewable silver-based amalgam film electrode Hg(Ag)FE is highly sensitive, reproducible, and linear, and is most effective in the negative potential range. There are many reasons not to use a classical mercury-based electrode, but the silver-based amalgam electrode seems to carry less risk of toxicity. The electrode can be refreshed mechanically before each measurement [39], and the electrode surface can be easily adjusted if necessary, which is an unquestionable advantage over a solid modified electrode. The electrode Hg(Ag)FE has already been successfully tested in highly sensitive assays for pharmaceuticals in various matrices [40,41,42].

Here, we develop a reliable, simple, rapid, low-cost, and highly sensitive method for BB determination that requires no extraction steps and is suitable for food matrices. The new procedure was examined and applied successfully to determine the BB content in several beverages and artificial fluids, and the results were compared to those of the classical spectrophotometric method.

## 2. Materials and Methods

### 2.1. Measurment Apparatus and Software

All voltammetric measurements were performed using a Multipurpose Electrochemical Analyzer (model M161) and an Electrode Stand (model M164), both manufactured by mtm-anko (Kraków, Poland). The classical three-electrode quartz cell has a volume of 10 mL. It consists of a homemade cylindrical silver-based mercury film electrode (Hg(Ag)FE), which was refreshed before each measurement. This electrode has a surface area of 1–14 mm^2^. The cell also contains a double-junction Ag/AgCl/KCl (3 M) reference electrode with a replaceable outer junction (3 M KCl), as well as a platinum wire auxiliary electrode (Mineral, Warszawa, Poland). Seven compact S220 pH meters (Mettler-Toledo, Greifensee, Switzerland) were used to perform pH measurements. Stirring was performed using a magnetic bar rotating at approximately 500 rpm. All experiments were carried out at room temperature. A spectrophotometer UV-VIS Cary 100 (Varian, Melbourne, Victoria, Australia) and an Elma ultrasonic bath (Schmidbauer GmbH, Gräfelfing, Germany) were used. Data processing and statistical calculations were performed in Statistica 13.3 (TIBCO Software Inc., Palo Alto, CA, USA) and OriginPro 2025 (OriginLab Corporation, Northampton, MA, USA).

### 2.2. Chemicals and Glassware

All reagents used were of analytical grade: acetic, boric, and phosphoric acids, sodium hydroxide, potassium nitrate, and chloride (Merck, Suprapur, Darmstadt, Germany); mercury GR (Guaranteed Reagent) for polarography (Merck, Darmstadt, Germany); Triton X-100 (Merck, Darmstadt, Germany); and citric acid (Sigma-Aldrich, Steinheim, Germany). The standard stock solutions of brilliant blue FCF (BB) (6.5 mg/5 mL) were prepared by dissolution of BB (Sigma-Aldrich, Steinheim, Germany) in quadruple-distilled water. Solutions with lower concentrations of BB were prepared by appropriate dilution of the stock solution. The silver substrate for the film electrode was prepared from 99.99% purity polycrystalline silver wire (⌀ 0.5 mm), sourced from Goodfellow Science Park in England. Before use, glassware was cleaned with a 1:10 aqueous solution of HNO_3_ (65%), followed by thorough rinsing with distilled water.

All the beverages were purchased from a local market. Artificial digestive juices (saliva, gastric juice) were prepared according to Jędrejko [43]. Artificial saliva (pH = 6.7) contained 15 mmol/L KHCO_3_, 2.5 mmol/L KH_2_PO_4_, 2.4 mmol/L Na_2_HPO_4_, 1.5 mmol/L CaCl_2_, 0.15 mmol/L MgCl_2_, and 0.15 mmol/L citric acid, dissolved in quadruple-distilled water, and the obtained solution did not contain enzymes naturally present in human saliva. Artificial gastric juice (pH = 2) was prepared in accordance with the Polish Pharmacopoeia, containing 3.2 g of pure pepsin and 2.0 g of NaCl dissolved in quadruple-distilled water. A total of 80 mL of 1 M HCl was added to adjust the pH, and quadruple-distilled water was added until a volume of 1 L was reached.

### 2.3. Standard Procedure of Measurements

The standard addition procedure and differential pulse voltammetry were used to perform the quantitative measurements. The procedure for renewing the mercury film Hg(Ag)FE electrode was carried out before each measurement. The electrode was conditioned after the refreshing step by applying a potential of −1350 mV (5 s). The working electrode, after being conditioned in this way, was used to determine brilliant blue FCF in the supporting potassium nitrate electrolytes (pH = 7), contained in a quartz voltammetric cell. The electrode potential was varied according to the following sequence: starting potential of −1300 mV for 5 s and a preconcentration step of −400 mV for 15 s. Then, differential pulse voltammograms were recorded in triplicate. The other experimental parameters were as follows: step potential, 6 mV; pulse potential, 30 mV; time step potential, 20 ms (10 ms of both waiting and probing time). Argon was used as an inert gas to carry out the measurements from deaerated solutions.

### 2.4. Analysis of Brilliant Blue FCF in Beverages

For the voltammetric analysis, 100 µL of diluted beverage was introduced directly into the voltammetric cell without extraction. Then, appropriate amounts of standard solutions were added in triplicate to the voltammetric cell. To remove dissolved gases, carbonated beverages were sonicated for 5 min., and those showing opacity were filtered through a 0.45 µm membrane. The above procedures were especially crucial for the BB spectrophotometric analysis. In the case of spectrophotometry, the absorption spectra were recorded in the range of 400–800 nm, and read at 629 nm (chosen λ_max_ wavelength). The appropriate regression equation was used to calculate the concentration of the compound determined.

## 3. Results

### 3.1. Effect of DPV Parameters on Brilliant Blue FCF Peak Characteristics

**Figure 1:**
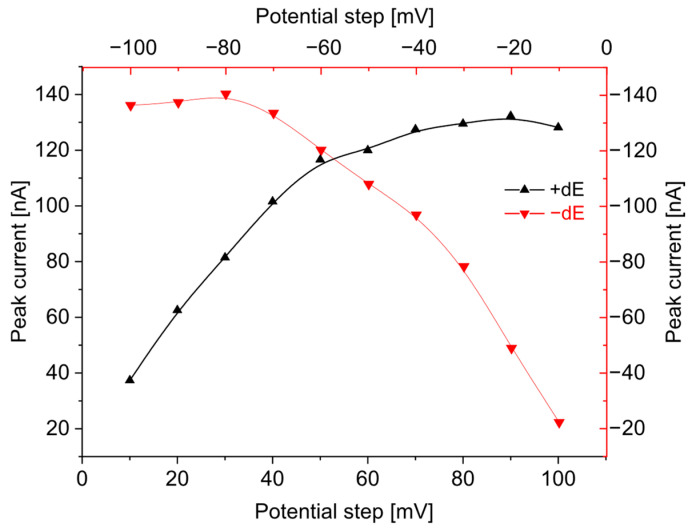
Dependence of pulse amplitude (from 10 mV to 100 mV) on peak current in negative (—) and positive (—) modes. Electrode surface area, 9.7 mm^2^. Instrumental parameters: E_s_ = 6 mV; t_p_, t_w_ = 20 ms; stirring rate, 500 rpm.

The key parameters of the DPV technique are pulse amplitude (dE), potential step amplitude (E_s_), and waiting (t_w_) and sampling time (t_p_). Measurements of BB in 0.1 M KNO_3_ were characterized by the formation of a sharp peak at a potential of −1072 mV. To optimize the instrumental parameters for the determination of brilliant blue FCF, the following variables were examined: differential potential (dE) ranging from −100 to 100 mV (in increments of 10 mV), step potential (E_s_) ranging between 1 and 20 mV, and pulse and waiting times (t_p_ and t_w_) ranging from 5 to 40 ms. At a pulse amplitude of 40 mV, the analyte’s peak current reached 99.48 nA and increased proportionally with higher amplitudes. However, amplitudes exceeding 40 mV resulted in a marked rise in background current without an efficient increase in the analytical signal of peak height; whenever amplitudes were above 90 mV, the peak current decreased. The decrease in pulse amplitude from −10 mV to −100 mV caused the peak potential to shift from −1036 mV to −958 mV (Figure 1). Considering the SNR (signal-to-noise ratio) value and shifting potential, the best results were obtained for an amplitude of 40 mV; the selected pulse amplitude was applied for further measurements.

**Figure 2:**
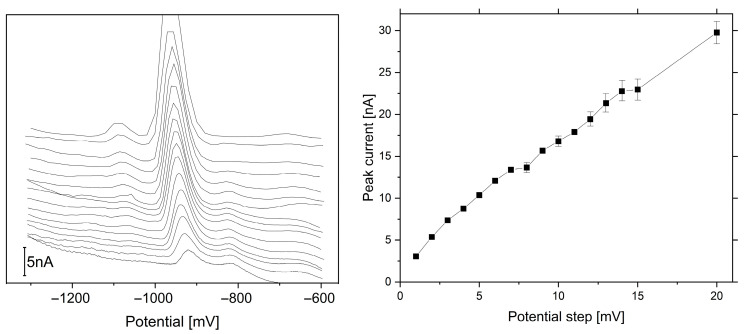
Dependence of potential pulse step (E_s_) on peak current for 1–20 mV. Instrumental parameters: ΔE = 40 mV; t_w_, t_p_ = 20 ms. Stirring rate, 500 rpm.

In the case of potential step amplitude, the BB peak current showed significant growth up to 20 mV with significant growth in the background current, a significant reduction in registered data, and, as a consequence, an increasingly poorly formed peak shape above 6 mV (Figure 2). A step potential of 6 mV was applied in further measurements based on the above. The waiting and probing times were varied within the range of 10–60 ms. The best results were obtained with a waiting time and probing time of 10 ms, so this value was chosen for further measurements. Two different constructions of mercury electrodes were selected for comparison in order to achieve a low detection limit for brilliant blue FCF.

**Figure 3:**
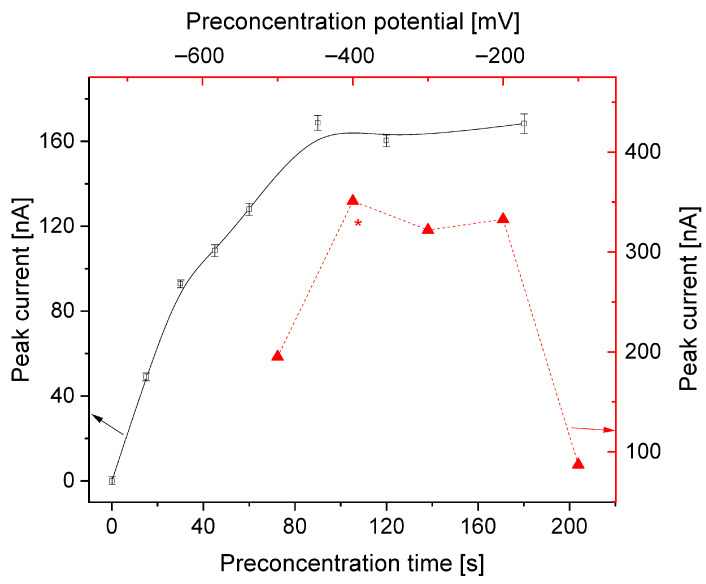
Dependence of preconcentration time and potential on peak current. Electrode surface area, 9.7 mm^2^. Instrumental parameters: dE = 40 mV; E_s_ = 6 mV; t_p_, t_w_ = 20 ms; stirring rate, 500 rpm. * This potential has been selected for further measurements in this study.

Another important element of DPV is the preconcentration step conditions, which include its potential and duration. In order to optimize this phase, the following parameters were checked: E_acc_ in the range of −100 to −500 mV, and t_acc_ in the range of 0–160 s. The best results were obtained using a −400 mV potential, whereas the peak current grew significantly with a longer accumulation time. The relationship between peak height and preconcentration time was characterised by two areas of linearity with a larger (0–30 s) and smaller slope (30–90 s) (Figure 3). A time of 15 s was selected for further analysis for practical reasons, but could be extended up to 90 s in the case of BB trace analysis.

The behaviour of the BB molecules on both the hanging mercury drop electrode (HMDE) and the renewable silver-based amalgam film electrode (Hg(Ag)FE) was studied. The comparison of the voltammograms obtained for these two electrodes is presented in Figure S1 (Supplementary Materials). The calculated current value of the BB peak, taking into account the different surface areas of electrodes, was 2.6 times higher for Hg(Ag)FE than for HMDE under the same conditions.

### 3.2. Effect of Type of Supporting Electrolyte and pH on Brillant Blue Peak

**Figure 4:**
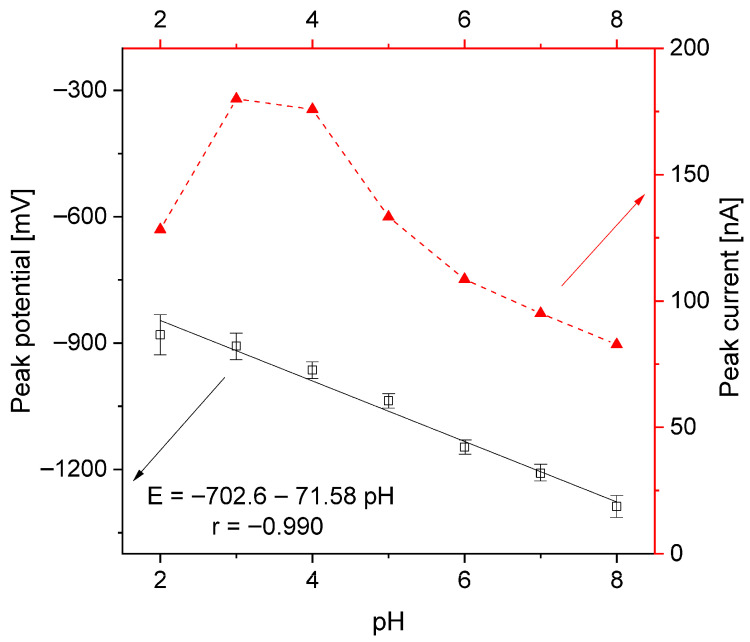
Dependence of peak current and potential on pH value. Instrumental parameters: ΔE = 40 mV; E_s_ = 6 mV; t_w_, t_p_ = 20 ms. Stirring rate, 500 rpm.

A variety of supporting electrolytes at different concentrations were evaluated as ionic media, including sodium hydroxide, acetic and phosphoric acids, neutral salts (KNO_3_, KCl), and buffers (Britton–Robinson, acetate, and phosphate). Potassium nitrate (V) was selected for subsequent analyses due to its favourable peak shape and high signal-to-background ratio. A concentration of 0.1 M was found to be optimal, offering both sufficient peak height and adequate ionic conductivity. The ideal pH range was determined to be between 3.0 and 4.0, as more acidic or alkaline conditions led to a reduction in peak current (Figure 4). The electrochemical reaction appears to involve the same number of electrons and protons, as the linear dependence of the peak current on the pH and the slope of 72 mV/pH are close to the theoretical value of 59 mV.

The pH also had an influence on the peak potential, which changed to positive values for lower pH values. In the investigated pH range, the peak potential shifted from −880 mV to −1288 mV and had a linear dependence on pH. However, taking into account the growing background current due to the hydrogen reduction, and the BB peak occurrence at a strong negative potential, a neutral electrolyte was considered. The best BB peak shape, a good signal-to-noise ratio (SNR), and optimal ionic medium conductivity were achieved for the solution of 0.1 M KNO_3_, which was applied for further measurements.

### 3.3. Influence of the Surface Area of the Hg(Ag)FE Electrode on Brilliant Blue FCF Peak

**Figure 5:**
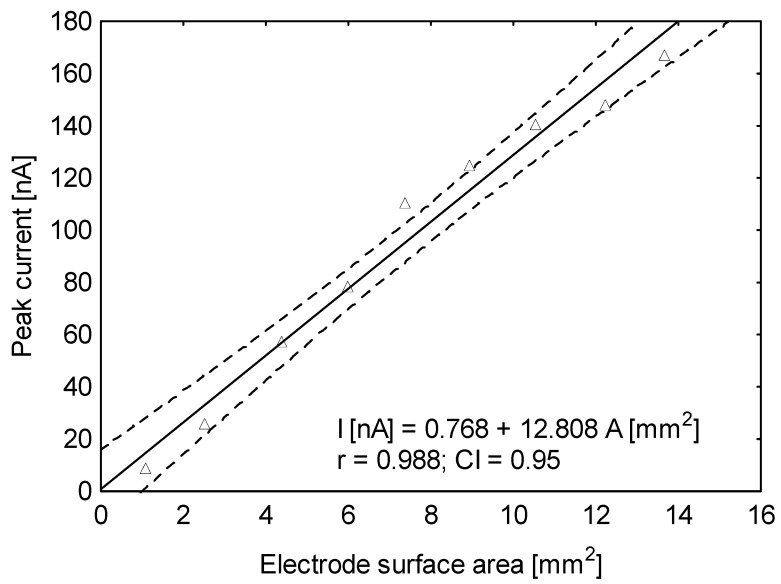
Dependence of brilliant blue FCF peak current on surface area of Hg(Ag)FE: 1.1–13.7 mm^2^ for 2 µg L^−1^ of brilliant blue FCF in 0.1 M KNO_3_. Instrumental parameters: ΔE = 40 mV; E_s_ = 6 mV; t_w_, t_p_ = 20 ms. Stirring rate, 500 rpm.

Solid electrodes typically offer a larger effective surface area than mercury drop electrodes. The Hg(Ag)FE electrode can be used to easily obtain a wide range of surface area changes on the working electrode, depending on the analytical requirements. So, in this case the surface area in the range of 0–13.9 mm^2^ was investigated: the BB peaks increased in size in proportion to the increase in the surface area of the working electrode (Figure 5). The parameters of the linear growth of the peak current vs. the surface of the working electrode are as follows: slope, 12.808 ± 0.241 [nA mm^−2^]; intercept, 0.768 ± 0.506 [nA]; and correlation coefficient r = 0.988. For further measurements, a 9.7 mm^2^ surface area was applied.

### 3.4. Analytical Performance

**Figure 6:**
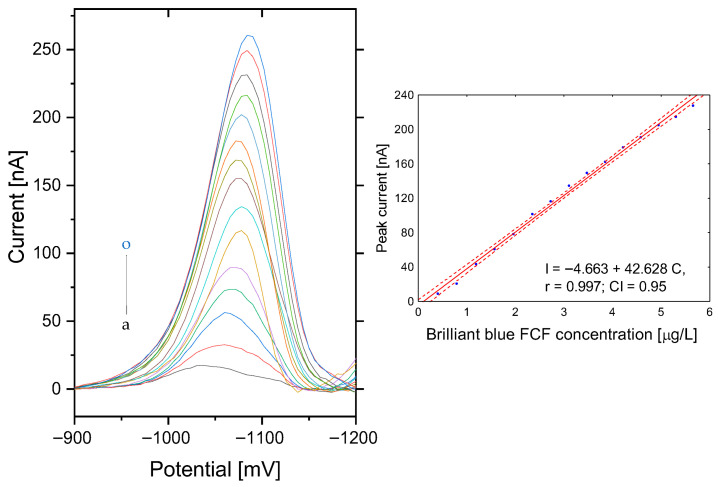
Linearity of voltammetric determination of brilliant blue FCF in the range of 0.72–5.7 µg L^−1^. The electrode area was 9.7 mm^2^. Instrumental parameters: ∆E = 40 mV; E_s_ = 6 mV; t_w_, t_p_ = 20 ms. Stirring rate, 500 rpm. Inset: dependence of brilliant blue FCF peak current on surface area. (The inset shows a calibration graph of peak current as a function of BB concentration with confidence interval bands).

Linearity was assessed up to 250 µg L^−1^; a narrower range was used for detailed evaluation. The differential pulse cathodic voltammograms of brilliant blue FCF for the 0.72–5.70 µg L^−1^ concentration range are presented in Figure 6. In this concentration range, the slope for the regression line is 42.628 ± 0.880 [nAµg^−1^ L], with a correlation coefficient of r = 0.997. The occurrence of autocorrelation of residues was assessed using the Durbin–Watson (D-W) test. In the case of BB, the value of the D-W statistic is d = 0.48, which is below the bottom threshold (d_l_ = 1.08; *p* = 0.05), so the autocorrelation of the random component was observed. Therefore, it was agreed that the effectiveness of the least squares estimators used to assess the linearity of the method would be reduced. The normality of the distribution of the residuals was examined by a Shapiro–Wilk test (W = 0.964 > W_0.05_ = 0.94; *p* = 0.88 > 0.05) and it was found that there is no basis for rejecting the hypothesis of a normal distribution of the random component. Therefore, heteroscedasticity of the random component was verified by Bartlett’s test, and the results were χ2 = 0.20, df = 1, and *p* = 0.65 > 0.05, which shows that the variance in the residuals in the model is constant.

The limits were estimated according to the following relationships: signal-to-baseline noise ratios of three and ten, respectively. For the short-time analysis, the obtained detection limit (LOD) of brilliant blue FCF—with a surface area of the working electrode of 10.9 mm^2^—and quantification limit (LOQ) were 0.24 µg L^−1^ and 0.72 µg L^−1^, respectively. At an analyte concentration of 2.0 µg/L, the method demonstrated a repeatability of 2.39% RSD across six successive measurements. The reproducibility of the method was very good, measured as RSD, with a value of 4.15%, and was calculated based on nine consecutive measurements on a fresh surface of working electrode.

Comparable or better analytical characteristics were obtained in comparison to those reported for other modified electrodes (Table 1). In most cases, a wide linear range was achieved at the expense of obtaining multiple linear segments with varying slopes, which inherently limits the practical application of such a dynamic range. In the present study, a linearity range of up to 250 µg L^−1^ was identified; however, we focused on a narrower interval, the characteristics of which have been described above. Considering the possibility of modifying the Hg(Ag)FE electrode surface area, a comparable wider range appears to be readily attainable. This approach offers a notable improvement in sensitivity over most methods currently available in the literature. However, it is mostly based on a longer adsorptive preconcentration (up to 500 s), which increases the measurement time and can lead to the co-preconcentration of other components of the sample. Moreover, as noted in the Introduction, the majority of methods compared in this study require significantly more intricate sensor preparation steps and often time-consuming electrochemical pretreatment. Nevertheless, the developed sensor is easier to fabricate, easier to regenerate (surface renewal processes), and more surface-reliable than solid electrodes, and it is also possible to use it outside of a laboratory setting.

Regarding measurement conditions, two pH options are most commonly used, neutral and acidic, with only one reported case employing alkaline conditions [30]. Neutral pH appears to be more justified, as above 6.82, the fully protonated form of BB microspecies is the predominant species, according to calculations using Marvin JS 24.3.0 (ChemAxon, Budapest, Hungary). This is another confirmation that the use of neutral pH is more justified, despite what is discussed mentioned in Section 3.2.

Accuracy was determined using beverage samples spiked with 4.5, 5.6, and 6.7 µg of BB. Samples were analysed according to the described procedure using the Hg(Ag)FE electrode described in Section 2.4. Analysis was performed using the standard addition method, and the recovery of BB ranged from 98.5 to 101.2% (Table 2), with results obtained for the final products (Table 3).

### 3.5. Interference

Beverages coloured with brilliant blue FCF, among other ingredients, contain sweeteners, active ingredients (caffeine, taurine), vitamins, acidity regulators, alcohol, and surface-active compounds, which are usually a source of strong interference in voltammetric methods. Ethanol, a nonionic surface-active compound (Triton X-100), citric acid, sodium benzoate, potassium citrate and sorbate, gum arabic, niacin, biotin, piridoxin, tartrazine, glucose, aspartame, and acesulfame K were investigated for this reason. The results showed that the investigated ingredients did not affect the brilliant blue FCF analysis. The quantitative effect of the surfactant Triton X-100 and solutions of artificial saliva and gastric juice on the determination of the selected dyes was also determined. It was found that Triton X-100 added at a concentration of 3.5 mg/L causes signal loss and limits the determination of BB. In the sample spiked with 20% artificial saliva, determination of brilliant blue was possible with relatively high accuracy (the recovery rate was not less than 82%). However, when spiked with 20% artificial gastric juice, the standard addition of BB was characterised by a non-linear increase in the height of the measured peaks. It is possible to determine BB, with lower efficiency of linear regression estimators, or to use a quadratic fitting model, obtaining a discrimination coefficient of r^2^ = 0.999.

## 4. Conclusions

The developed method of differential cathodic voltammetry enables the determination of Brilliant Blue FCF even in trace amounts using a cylindrical electrode with a silver-based mercury coating (Hg(Ag)FE), which is renewed before each measurement. The method demonstrates high reproducibility, with a relative standard deviation (RSD) of 1.6%, based on measurements conducted on freshly prepared electrode surfaces. The method demonstrated good precision (RSD = 2.39%), high sensitivity (LOD = 0.24 µg L^−1^, LOQ = 0.72 µg L^−1^), and a linearity range of 0.72–5.6 µg L^−1^. Due to its high sensitivity, the technique remains effective even in the presence of commonly co-occurring dyes. The obtained results confirm that the proposed method and Hg(Ag)FE may be incorporated into future portable, out-of-laboratory sensor systems. Fortunately, none of the samples exceeded the acceptable levels of dyes. Quantitative analysis performed using the proposed method confirmed that the levels of brilliant blue FCF present in the analysed beverages do not exceed the thresholds established by food safety regulations. The obtained results not only verify that BB levels are within acceptable limits, but also enrich the limited data on the quantitative compositions of ‘popular’ beverages.

## Figures and Tables

**Table 1:** Comparison of DPV detection of BB using different working electrodes.

Electrode Type	Electrolyte (pH)	Linearity Range	LOQ [µM]	LOD [µM]	Ref.
CuNiFe_2_O_4_	PBS (3)	5–700 **	5.3	1.6	[36]
CI/CBPCE	BR (10)	0.025–2.5	4 × 10^−2^	1 × 10^−2^	[30]
MnO_2_/NBE/GCE	PBS (7)	0.25–2.5; 2.5–15	0.1	4 × 10^−2^	[33]
MWCNT/CPE	PBS (2)	0.5–0.8; 0.8–25	0.3	8 × 10^−2^	[32]
MWCNT/GO-RTIL/GCE	BR (2)	0.008–0.01; 0.01–0.1	1 × 10^−2^	4 × 10^−3^	[34]
IL-EGPE *	BR (2)	0.005–4 **	6 × 10^−3^	2 × 10^−3^	[31]
MoS_2_/PVP/GCE	PBS (7)	0.8–209; 255–1150	3 × 10^−2^	9 × 10^−3^	[35]
Ag(Hg)FE	KNO_3_ (7)	0.7–5.6	1 × 10^−3^	3 × 10^−4^	This work
CV/CuONPs/CPE	KCl (7); PBS (3)	1 × 10^−5^–1 × 10^−3^	1 × 10^−4^	3 × 10^−5^	[37]

* In this case square-wave voltammetry was used; ** two linear ranges with different slope; PBS—phosphate buffer; BR—Britton–Robinson buffer; CI/CBPCE—carbon ink/carbon black–polyethylene composite electrode; MnO_2_/NBE/GCE—MnO_2_-nanorod-based electrode/glassy carbon electrode; MWCNT/CPE—multi-walled carbon nanotube/carbon paste electrode; MWCNT/GO-RTIL/GCE—multi-walled carbon nanotube/graphite oxide-room temperature ionic liquids/glassy carbon electrode; IL-EGPE—ionic liquid-expanded graphite composite paste electrode; MoS_2_/PVP/GCE—MoS_2_/polyvinylpyrrolidone/glassy carbon electrode; CV/CuONPs/CPE—poly (crystal violet)/CuO nanoparticles/carbon paste electrode.

**Table 2:** Recovery of BB determination in 4MOVE sample.

	Added [µg]	Found [µg]	Recovery [%]
Brilliant Blue FCF	-	5.60 ± 0.24	-
	4.5	10.22 ± 0.31	101.2
	5.6	11.03 ± 0.28	98.48
	6.7	12.28 ± 0.33	99.84

**Table 3:** Results of E133 content of different samples detected by DPV and UV-Vis methods.

Sample	DPV [µg mL^−1^]	UV-Vis [µg mL^−1^]	F-Test	*t*-Test
4MOVE	5.60 ± 0.24	5.27 ± 0.11	0.40	<0.05 *
Garage	8.28 ± 0.19	8.41 ± 0.38	0.20	0.20
Izzy Kamikaze	15.16 ± 0.35	15.39 ± 0.42	0.57	0.24
Mirinda	16.18 ± 0.32	16.10 ± 0.41	0.64	0.38
drWitt	1.99 ± 0.03	1.92 ± 0.01	0.18	<0.05 *
Step’On	4.04 ± 0.11	4.09 ± 0.10	0.93	0.27
IsoFresh	3.85 ± 0.10	3.82 ± 0.11	0.78	0.47
Artificial saliva	1.97 ± 0.04 (95.16) **	1.99 ± 0.04 (96.14)	0.77	0.47
Gastric juice	1.73 ± 0.06 (83.57)	1.38 ± 0.05 (66.67)	0.86	<0.05 *

* Differences were statistically significant; ** the recovery (%) result is shown in brackets.

## Data Availability

The data is available from the authors upon reasonable request.

## Supplementary Materials

The following supporting information can be downloaded at: https://www.mdpi.com/article/10.3390/s25206424/s1.
