# Supplementary material for: A Reliable and Simple Voltammetric Method for Analysis of Brilliant Blue FCF
Source: Sensors (Basel). 2025 Oct 17;25(20):6424. doi: 10.3390/s25206424 (PMC12567795; doi:10.3390/s25206424)
Supplement: Supplementary file 1 [file sensors-25-06424-s001.zip › sensors-3906927-supplementary.docx]

Supplementary materials

A Reliable And Simple Voltammetric Method For Analysis Of Brilliant Blue FCF

Marek Szlósarczyk^1^, Robert Piech^2^, Bartłomiej Pach^1^, Mariusz Stolarczyk^1^, Urszula Hubicka^1^

**Figure S1.** Comparison of voltamograms obtained for 2.0 µgmL-1 BB in 0.1 M KNO3 for HMDE and Hg(Ag)FE electrode. The electrode surface areas were 5.7 mm2, 1.6 mm2 for the (Hg(Ag)FE) and HMDE, respectively. Instrumental parameters: ΔE = 40 mV, Es = 6 mV, tw, tp = 20 ms. Stirring rate, 500 rpm.

**Figure S2.** Series of voltamograms obtained for 2.0 µgmL^-1^ BB in 0.1 M KNO_3_ on Hg(Ag)FE electrode with different preconcentration time 0-180 s at potential -800 mV vs Ag/AgCl. Instrumental parameters: ΔE = 40 mV, Es = 6 mV, tw, tp = 20 ms. Stirring rate, 500 rpm.

**Figure S3.** Series of voltamograms obtained for 2.0 µgmL^-1^ BB in 0.1 M KNO_3_ on Hg(Ag)FE electrode with different potential step amplitude from -100 mV to 100 mV. Instrumental parameters:, Es = 6 mV, tw, tp = 20 ms. Stirring rate, 500 rpm.

**Figure S4.** Typical voltammograms of BB obtained for artificial saliva in 0.1 M KNO_3_ on the (Hg(Ag)FE). Instrumental parameters: ΔE = 40 mV, Es = 6 mV, tw, tp = 20 ms. Stirring rate, 500 rpm.

**Figure S5.** Typical voltammograms of BB obtained for beverage sample in 0.1 M KNO_3_ on the (Hg(Ag)FE). Instrumental parameters: ΔE = 40 mV, Es = 6 mV, tw, tp = 20 ms. Stirring rate, 500 rpm.
